# Role of glutathione and cysteine in acrylamide metabolism during *in vitro* and *in vivo* digestion

**DOI:** 10.1002/jsfa.70571

**Published:** 2026-03-12

**Authors:** Burçe Ataç Mogol, Choong‐In Yun, Aytül Hamzalıoğlu, Selin Çakmak, Yeşim Şensoy, Nazmiye Çitler, Eunjung Lee, Shin Young Park, Junwon Byun, Young‐Jun Kim, Sanguine Byun, Vural Gökmen

**Affiliations:** ^1^ Food Quality and Safety (FoQuS) Research Group, Department of Food Engineering Hacettepe University Ankara Turkey; ^2^ Department of Food Science and Biotechnology Sungkyunkwan University Suwon Republic of Korea; ^3^ Research Institute of Food and Biotechnology Seoul National University of Science and Technology Seoul Republic of Korea; ^4^ Research Group of Traditional Food Korea Food Research Institute Wanju Republic of Korea; ^5^ Department of Biotechnology, College of Life Science and Biotechnology Yonsei University Seoul Republic of Korea; ^6^ Department of Food Science and Biotechnology Seoul National University of Science and Technology Seoul Republic of Korea

**Keywords:** acrylamide, potato chips, glutathione, cysteine simulated digestion, mice model, urinary acrylamide biomarkers

## Abstract

**Background:**

Acrylamide, a probable human carcinogen found in thermally processed potato products, is reactive towards amino and thiol compounds. This reactivity suggests acrylamide might react with them in the gastrointestinal tract resulting in mitigation of associated risk with acrylamide. This study investigated the impact of the thiol compounds, glutathione and cysteine, on acrylamide fate in potato chips both *in vitro* and *in vivo*.

**Results:**

Acrylamide levels in control potato chips declined significantly during simulated digestion, with 35% and 57% reductions in the gastric and in the bioaccessible fraction, respectively. Incorporating thiol compounds (1% glutathione or 0.5% cystine) lowered initial acrylamide formation (*P* < 0.05) but did not further eliminate acrylamide during digestion (*P* > 0.05). In contrast, co‐digestion of potato chips with free thiols enhanced acrylamide elimination, with 0.5% cysteine or 1% glutathione achieving a 19% reduction after the oral phase. With 1% glutathione, acrylamide elimination reached 49% in the gastric phase and 70% in the intestinal phase, while 0.5% cysteine achieved 67% elimination in the gastric phase. Male C57BL/6N mice were fed diets supplemented with reformulated potato chips containing glutathione and cysteine for 7 days, and urinary biomarkers (glycidamide, AAMA, and GAMA) were analyzed. Control diets with regular potato chips led to elevated acrylamide metabolites (AAMA, GAMA), while cysteine‐containing chips reduced both. Glutathione‐containing chip consumption resulted in lower GAMA but higher AAMA, suggesting enhanced acrylamide detoxification.

**Conclusion:**

The reformulation of potato chips with thiol compounds, or co‐consumption of thiol compounds with acrylamide‐rich foods, can effectively mitigate the potential toxicological effects of acrylamide. © 2026 The Author(s). *Journal of the Science of Food and Agriculture* published by John Wiley & Sons Ltd on behalf of Society of Chemical Industry.

**Abbreviations**
AAMA: (*N*‐acetyl‐*S*‐(2‐carbamoylethyl)‐l‐cysteine
AAMA‐sul: *N*‐acetyl‐*S*‐(2‐carbamoylethyl)‐l‐cysteine sulfoxide
Acr: acrylamide
Ala: alanine
Arg: arginine
Asp: aspartic acid
CE: collision energy
ESI: electrospray ionization
GAMA: *N*‐(*R*,*S*)‐acetyl‐*S*‐(2‐carbamoyl‐2‐hydroxyethyl)‐l‐cysteine
Gln: glutamine
Glu: glutamic acid
Gly: glycine
GSH: glutathione
GSSG: oxidized glutathione
His: histidine
HPLC: high‐performance liquid chromatography
iso‐GAMA: *N*‐acetyl‐*S*‐(1‐carbamoyl‐2‐hydroxyethyl)‐l‐cysteine
LC‐HRMS: liquid chromatography with high‐resolution mass spectrometry
LOD: level of detection
LOQ: level of quantification
*m*/*z*
: mass‐to‐charge ratio
Met: methionine
MS: mass spectrometry
Phe: phenylalanine
Pro: proline
PTFE: polytetrafluoroethylene
Ser: serine
Thr: threonine
Tyr: tyrosine
UHPLC: ultrahigh‐performance liquid chromatography

## INTRODUCTION

The Maillard reaction is a chemical reaction that occurs between amino compounds, including free amino acids, peptides and proteins, and carbonyl compounds, such as reducing sugars. It is observed in all biological systems including the human body and is related to many pathological disorders.^1^ The Maillard reaction is also responsible for the nonenzymatic browning and flavor development of foods during heating.^2^ On the other hand, undesirable compounds are formed during the Maillard reaction that occur due to the processing of foods at elevated temperatures. Among these, acrylamide is particularly noteworthy due to its toxicity.^3^, ^4^, ^5^ Acrylamide is classified as a ‘probable human carcinogen’ in Group 2A.^6^ Potato‐based food products are among the primary sources of exposure to acrylamide.^7^ Therefore, mitigating acrylamide formation in certain food products, such as potato products, is of importance. Numerous efforts have been made to control acrylamide formation in heat‐processed foods being published as Acrylamide Toolbox by FoodDrinkEurope.^8^ On the other hand, addressing the potential health risks associated with acrylamide exposure remains a critical concern.

There are limited data about the fate of acrylamide during digestion. Thiol compounds were found to be the most efficient scavengers of acrylamide during *in vitro* digestion.^4^ Accordingly, a significant portion of acrylamide disappeared at the end of the intestinal phase during *in vitro* digestion of thermally processed foods.^4^ Acrylamide is an electrophilic molecule, being an *α*,*β*‐unsaturated carbonyl compound, making it quite accessible for the reactions with thiol compounds and amino acids through Michael addition. In biological and food systems, sulfur‐containing groups are the primary nucleophiles, making the thiol groups of cysteine highly prone to forming acrylamide adducts. Acrylamide can also react with other nucleophilic sites, such as the amino group of lysine, the imidazole group of histidine and the amino groups of free amino acids, leading to various Michael adducts.^9^ Similar results were also observed with other Maillard reaction products with an electrophilic nature, indicating they were scavenged by the food components released during digestion.^10^ The decrease of acrylamide by the interactions with nucleophiles under alkaline intestinal pH was included in the review by Hamzalıoğlu and Gökmen.^11^ This was due to the interactions of acrylamide with amino and thiol compounds released during the digestion of foods. All these aspects might be important for the reformulation of diet plans such that consumption of acrylamide‐rich potato chips together with scavengers might be effective in controlling the hazardous effects of acrylamide in the gastrointestinal tract.

Our previous findings indicated that thiol compounds, such as glutathione (GSH) and cysteine, were effective in the elimination of acrylamide in reformulated potato chips.^12^ As thiol compounds, GSH and cysteine are commonly found in a variety of foods, including cereal products, fresh fruits, vegetables and raw meats.^13^ Furthermore, they have recently gained widespread attention as a dietary supplement. Regarding their reactivity towards acrylamide, co‐consumption of thiol compounds with acrylamide‐containing foods presents promise for mitigating the adverse effects associated with acrylamide.

Following absorption from the gastrointestinal tract, acrylamide undergoes rapid and extensive biotransformation into glycidamide, a more toxic and active metabolite.^14^ Urinary excretion of acrylamide and metabolites provides useful biomarkers for monitoring acrylamide detoxification and conversion to glycidamide. Mercapturic acid conjugates of acrylamide and glycidamide, including four mercapturic acid adducts (*N*‐acetyl‐*S*‐(2‐carbamoylethyl)‐l‐cysteine (AAMA), *N*‐acetyl‐*S*‐(2‐carbamoylethyl)‐l‐cysteine sulfoxide (AAMA‐sul), *N*‐(*R,S*)‐acetyl‐*S*‐(2‐carbamoyl‐2‐hydroxyethyl)‐l‐cysteine (GAMA) and *N*‐acetyl‐*S*‐(1‐carbamoyl‐2‐hydroxyethyl)‐l‐cysteine (iso‐GAMA)), have been found in urine. These conjugates and glycidamide might be useful for predicting the presence of acrylamide.^15^ Existing studies on the *in vivo* effects of naturally available bioactive compounds on acrylamide metabolism in actual food systems are limited. The mechanism of action of targeted compounds on acrylamide needs to be elucidated both *in vitro* and *in vivo* to provide more scientific evidence on their potential use as food additives and dietary supplements by the food industry.

The primary aim of the study reported here was to investigate how selected thiol compounds, i.e. GSH and cysteine, affect the fate of acrylamide in potato chips after consumption. To achieve this, the study uses two complementary approaches: (i) *in vitro* simulated digestion experiments to evaluate changes in acrylamide during gastrointestinal digestion and (ii) *in vivo* studies in a mouse model to determine the influence of these thiol compounds on the formation and excretion of acrylamide‐derived urinary biomarkers.

## MATERIALS AND METHODS

### Materials

Agria potatoes were obtained from a local producer in Ankara. Waxy starch was kindly provided by Farin Kimya (İzmir, Türkiye). Food‐grade GSH was purchased from Pure Encapsulations (Nestle Health Science, USA) and food‐grade cysteine (l‐cysteine hydrochloride anhydrous) was purchased from Smart Kimya Tic. Ve Dan. Ltd Şti. (İzmir, Türkiye).

Acrylamide (>99%), acrylamide‐*d*_3_, l‐glutathione reduced (>98%), l‐cysteine (>98.5%), *α*‐amylase from porcine pancreas (≥10 U mg^−1^ solid), pepsin from porcine gastric mucosa (≥250 U mg^−1^ solid), pancreatin from porcine pancreas (8 × USP), ammonium bicarbonate, sodium bicarbonate, sodium carbonate, bile extract and formic acid were purchased from Sigma‐Aldrich (St Louis, MO, USA). Analytical‐grade zinc sulfate (Carrez I), potassium hexacyanoferrate (Carrez II), sodium hydroxide and calcium chloride were obtained from Merck (Darmstadt, Germany). Glycidamide, AAMA and AAMA‐*d*_3_ were obtained from Toronto Research Chemicals (Toronto, ON, Canada). GAMA was supplied by TLC Pharmaceutical Standards (Newmarket, Ontario, Canada). Acrylamide‐*d*_3_ and AAMA‐*d*_3_ were employed as internal standards to ensure analytical accuracy and precision. HPLC‐grade water and acetonitrile were purchased from JT Baker (Phillipsburg, NJ, USA). Nylon syringe filters (0.45 μm) were obtained from Isolab Laborgeräte GmbH (Wertheim, Germany), and Oasis MCX SPE cartridges were purchased from Waters Corp. (MA, USA).

### Preparation of reformulated potato chips

Potato flour was obtained by freeze‐drying of potatoes. The recipe and the preparations of the potato chips were done according to Mogol *et al*.^12^

The control recipe was composed of potato flour (25.0 g), waxy starch (2.94 g), sucrose (0.5 g), salt (0.34 g) and water (31.0 mL). GSH (1.0%) or cysteine (0.5%) was dissolved in water to include in the reformulated chip recipes.

Firstly, all the dry ingredients were combined, then water was added and blended in a Kitchen Aid 5KSM150 (MI, USA) mixer in three stages: first stage for 1 min, second stage for 45 s and third stage for 45 s. Then, the dough was rolled into a thin layer using the pasta roller attachment to obtain the dough with the same thickness. Flattened dough was smoothly cut into 3 cm circles and the pieces weighing between 0.87 and 0.94 g were selected for further thermal processing to obtain reproducible chips. Prior to baking, low‐temperature drying was done to achieve homogeneous baking at high temperatures. The chips were baked at 175 °C for 3 min in an oven (Memmert UNE 400, Germany) after being dried at 90 °C for 15 min in an oven (Nüve FN 400, Türkiye) to reach same moisture levels. All the chips were powdered and kept at −20 °C for further analysis.

### *In vitro* digestion of potato chips

**Figure 1:**
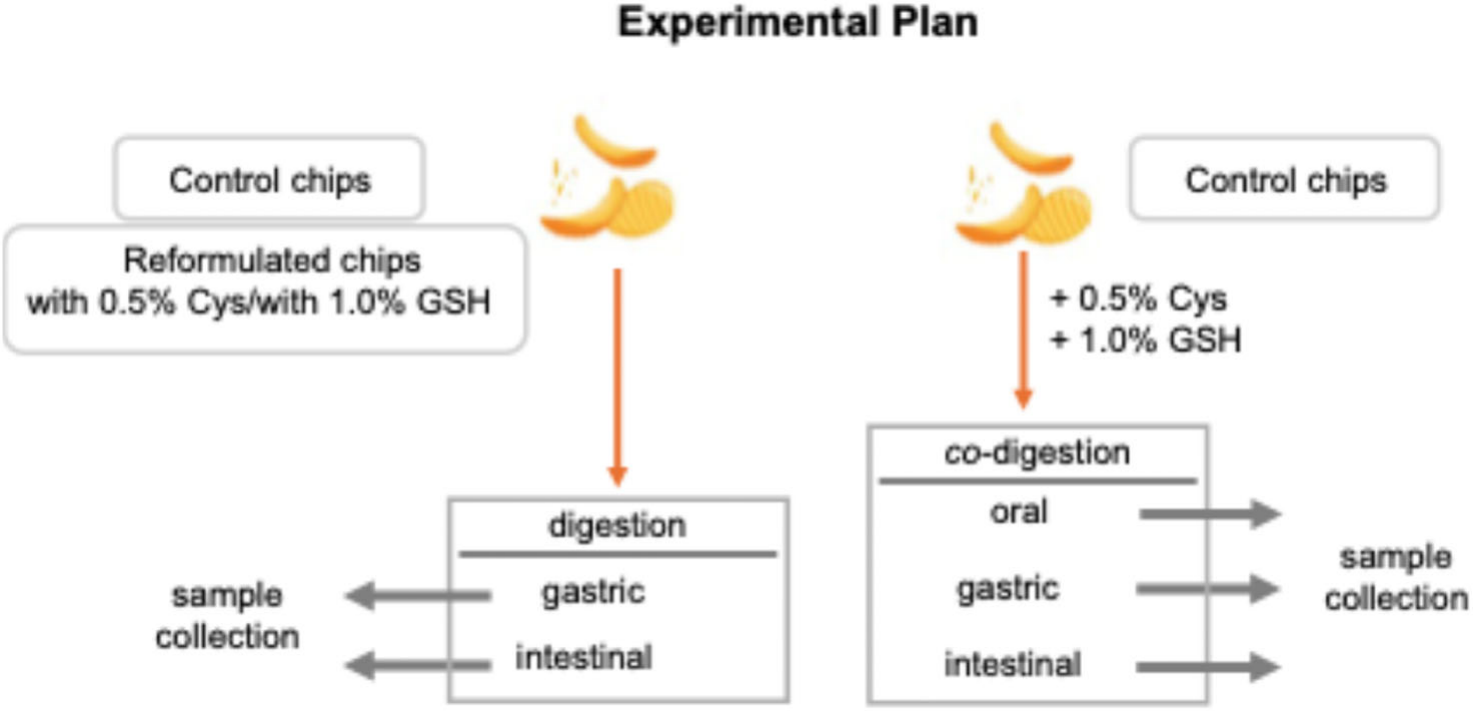
Experimental plan for digestion and co‐digestion experiments (Cys, cysteine; GSH, glutathione).

To evaluate how thiol compounds influence the fate of acrylamide during digestion, two experimental approaches were used: First, reformulated potato chips containing either 0.5% cysteine or 1% GSH were subjected to *in vitro* digestion. Second, regular potato chips were mixed with 0.5% cysteine or 1% GSH solutions and then immediately digested *in vitro* to simulate co‐digestion (Fig. 1). For the preparation of co‐digestion samples, firstly, 0.5% cysteine solutions as well as 1% GSH solutions were prepared freshly in water. Control potato chip samples (500 mg) were suspended in 1 mL of cysteine or GSH solutions. For *in vitro* digestion, a standardized INFOGEST protocol including oral, gastric and intestinal phases was used.^16^ At the end of the gastric phase, digests were centrifuged to separate the supernatant, to obtain the ‘gastric phase’ fraction. Intestinal phase as well as bioaccessible fraction refers to the supernatant (aqueous phase) containing soluble and potentially absorbable compounds after intestinal digestion. Samples at the end of the oral phase were also collected for co‐digestion experiments.

### Determination of acrylamide in potato chips and *in vitro* digesta samples

A multiple‐stage extraction technique was employed to extract acrylamide from reformulated potato chips, as previously described.^17^ Following the extraction, a clean‐up procedure was applied to both potato chip extracts and aliquots collected after gastric phase and intestinal phase of the *in vitro* digestion process. Acrylamide was analyzed using an Agilent 6475 Triple Quadrupole MS coupled to an Agilent 1290 Infinity II HPLC in ESI positive mode according to Mogol *et al*.^12^ A calibration curve for quantification of acrylamide was built at concentrations of 1, 2, 5, 10, 20 ng mL^−1^. The LOD and LOQ were 0.3 and 0.9 ng mL^−1^, respectively.

### Determination of acrylamide–thiol adducts in potato chips and *in vitro* digesta samples

To analyze the adducts of acrylamide using liquid chromatography with high resolution mass spectrometry (LC‐HRMS), the aliquots obtained at the end of the digestion of potato chips, which is the bioaccessible fraction, were diluted with acetonitrile (50:50, v/v). Similarly, aqueous extracts of potato chips were diluted with acetonitrile (50:50, v/v). Clarification and the analysis of the digests for certain acrylamide adducts were done using HRMS according to Mogol *et al*.^12^ Corresponding ions of adducts of acrylamide were extracted from the total ion chromatograms. The list of *m*/*z* values monitored are presented in Table 1.

**Table 1:** Values of *m/z* and chemical formulas of acrylamide (Acr) adducts, thiols and oxidized thiols monitored by LC‐HRMS in potato chips and bioaccessible fraction of their digesta

Amino acid adduct	Chemical formula	[M + H]
Ala‐Acr	C_6_H_12_N_2_O_3_	161.09207
Arg‐Acr	C_9_H_19_N_5_O_3_	246.15607
Asp‐Acr	C_7_H_12_N_2_O_5_	205.08190
Gln‐Acr	C_8_H_15_N_3_O_4_	218.11353
Glu‐Acr	C_8_H_14_N_2_O_5_	219.09755
Gly‐Acr	C_5_H_10_N_2_O_3_	147.07642
His‐Acr	C_9_H_14_N_4_O_3_	227.11387
Thr‐Acr	C_7_H_14_N_2_O_4_	191.10263
Met‐Acr	C_8_H_16_N_2_O_3_S	221.09544
Phe‐Acr	C_12_H_16_N_2_O_3_	237.12337
Pro‐Acr	C_8_H_14_N_2_O_3_	187.10772
Ser‐Acr	C_6_H_12_N_2_O_4_	177.08698
Tyr‐Acr	C_12_H_16_N_2_O_4_	253.11828
Other adducts/compounds		
2‐Acr‐cysteine	C_9_H_17_N_3_O_4_S	264.10125
Acr‐GSH	C_13_H_22_N_4_O_7_S	379.1282
GSH	C_10_H_17_N_3_O_6_S	308.09108
GSSG	C_20_H_32_N_6_O_12_S_2_	613.15924
Cysteine	CHNOS	122.02703
Cystine	C_6_H_12_N_2_O_4_S_2_	241.03112

### *In vivo* studies

Six‐week‐old male C57BL/6N mice were obtained from Koatech Co. (Pyeongtaek, South Korea). The mice were housed at a constant temperature (21–25 °C) and humidity (50–60%) under a 12 h light/dark cycle with free access to food and water. After a one‐week adaptation period, the mice were randomly divided into four groups (*n* = 5 or *n* = 7 per group). The standard group was provided with a standard diet (Teklad Global 18% Protein Rodent diet), while the control group received a diet containing control potato chips. The 1% GSH group was fed a diet supplemented with potato chips containing 1% GSH, and the 0.5% cysteine group received a diet supplemented with potato chips containing 0.5% cysteine. All experimental diets were formulated by incorporating the respective potato chip variations into standard laboratory chow, with potato chips comprising 15% of the total diet. Diets with or without food containing potato chips and drinking water were provided *ad libitum* for 7 days. Urine samples were collected from mice housed individually in metabolic cages (Techniplast) for a 4 h period, during which no additional food was provided. The samples were subsequently stored at −80 °C until further analysis. At the end of the experiment, the mice were sacrificed under isoflurane anesthesia via inhalation.

### Analysis of acrylamide biomarkers in urine samples

The pretreatment of mouse urine was modified from a previous study.^5^ An aliquot of thawed mouse urine (100 μL) was spiked with 100 μL of a mixed internal standard solution (AAMA‐*d*_3_) dissolved in 90% acetonitrile in 0.2% (v/v) formic acid at a concentration of 1 μg mL^−1^. To this, 900 μL of acetonitrile in 0.2% (v/v) formic acid was added, and the mixture was vortex‐mixed for 30 s. The resulting solution was then filtered through a 0.22 μm PTFE hydrophilic syringe filter and prepared for injection into the LC–MS/MS system.

Glycidamide, AAMA and GAMA were quantified using a Thermo Scientific Vanquish UHPLC system coupled with a TSQ Quantis mass spectrometer (Thermo Fisher Scientific, Waltham, MA, USA). Chromatographic separation was performed on a Kinetex HILIC column (2.6 μm, 2.1 mm × 100 mm; Phenomenex, Torrance, CA, USA). The mobile phase consisted of an isocratic 4:96 (v/v) mixture of 0.2% formic acid in water (A) and 0.2% formic acid in acetonitrile (B). The injection volume was set to 5 μL, with a flow rate of 0.3 mL min^−1^, and the column temperature was maintained at 30 °C throughout the analysis. Each chromatographic run lasted for 5 min. The source parameters were set to operate in positive ion mode (ESI^+^) with the following conditions: sheath gas flow rate at 50 arbitrary units, auxiliary gas at 10 arbitrary units, sweep gas at 1 arbitrary unit, spray voltage at 3500 V, vaporizer temperature at 350 °C and ion transfer tube temperature at 300 °C. These source settings were fine‐tuned during the method development phase. Data collection was carried out using multiple reaction monitoring mode. The cone voltage and collision energy (CE) were adjusted for each transition, and two product ions (one quantifier and one qualifier) were monitored for glycidamide, AAMA and GAMA. The selection of ions for the target compounds was modified based on references to previous studies.^18^, ^19^, ^20^ Glycidamide, AAMA and GAMA were quantified using AAMA‐*d*_3_ as the internal standard. The precursor ion was [M + H]^+^ 88 and product ions were *m*/*z* 43.9 (CE of 16 V) and *m*/*z* 73.9 (CE of 7 V) for glycidamide, the precursor ion was [M + H]^+^ 235 and product ions were *m*/*z* 103.9 (CE of 22 V) and *m*/*z* 193 (CE of 11 V) for AAMA, the precursor ion was [M + H]^+^ 251 and product ions were *m*/*z* 119.9 (CE of 22 V) and *m*/*z* 145.9 (CE of 14 V) for GAMA and the precursor ion was [M + H]^+^ 238 and the product ion was *m*/*z* 192 (CE of 10 V) for AAMA‐*d*_3_. Thermo Xcalibur™ 3.1 and TraceFinder 4.1 software (Thermo Fisher Scientific, San Jose, CA, USA) were employed for data acquisition and quantification.

The method validation was conducted following the guidelines of the Association of Official Analytical Chemists and the International Conference on Harmonization Q2(R1).^21^, ^22^ The matrix effect was calculated by taking the slope of the calibration curve obtained from the matrix‐matched sample and dividing it by the slope of the calibration curve obtained from the pure solvent. The result was then subtracted by one and multiplied by 100 to express the matrix effect as a percentage. The matrix effect values were 12.34% for glycidamide, 5.76% for AAMA and 3.48% for GAMA, indicating minimal matrix interference. By matrix‐matched calibration, the linearity of the method was good, with *R* ^2^ values ranging from 0.9993 to 1.0000 for all compounds. LOD values for glycidamide, AAMA and GAMA were 0.16, 0.0016 and 0.0016 μg mL^−1^, respectively. LOQ was estimated three times the LOD value.

Accuracy and precision were tested by spiking artificial urine with mixed standard solutions. The accuracy for glycidamide ranged from 96.74 ± 9.14% to 110.75 ± 7.01%. The accuracies for AAMA and GAMA were found to be between 98.30 ± 2.04% and 103.66 ± 2.04% and between 94.38 ± 3.87% and 111.16 ± 4.62%, respectively. The precision ranged from 0.68% to 9.45%, confirming that the method meets the AOAC validation criteria.^21^

### Ethical statement

All animal experiments were conducted in accordance with the procedures approved by the Korea Food Research Institute Animal Care and Use Committee (KFRI‐M‐24071).

### Statistical analysis

Statistical analysis of the data was conducted using analysis of variance (ANOVA) and a Duncan *post hoc* test with a 95% significance level. The statistical software XLStat (Lumivero, France) was used.

## RESULTS AND DISCUSSION

### Changes in acrylamide concentration during *in vitro* digestion of reformulated chips

Acrylamide is a concern in potato products, which are among the most significant sources of exposure. Consumption of acrylamide‐rich potato chips together with scavengers is a potential strategy in controlling the hazardous effects of acrylamide in the gastrointestinal tract. This study investigated the possible elimination of acrylamide in the gastrointestinal tract in the presence of thiol compounds.

Control and reformulated chips, containing 0.5% Cys or 1% GSH, were digested according to INFOGEST protocol including oral, gastric and intestinal phases. The reformulated potato chips contain thiols embedded within the matrix, which imposes matrix effects that limit the immediate release of both acrylamide and thiol compounds into the bolus, making such reactions less likely to occur at this stage. For this reason, acrylamide content only after gastric and intestinal phase from digestion of reformulated chips were reported (Table 2). The initial acrylamide concentration of control chips before digestion was 1749.6 ± 0.1 μg kg^−1^. This concentration was relatively high considering the benchmark levels (750 μg kg^−1^) in potato products.^23^ A significant reduction in acrylamide levels was observed after both gastric and intestinal phases. The acrylamide elimination was 35% in the gastric phase, whereas in the bioaccessible fraction (after intestinal phase), 57% of acrylamide was eliminated in the control potato chips. Similar reductions in the intestinal phase of *in vitro* simulated digestion were also observed in our previous findings.^4^ Within that study, acrylamide levels significantly decreased in various types of biscuits and potato products. In digested potato fries, the reduction reached up to 97%, while in sweet and non‐sweet biscuits, it was between 37% and 70% by the end of the intestinal phase. Similarly, a previous study reported that acrylamide levels decreased by approximately 72% in potato chips and about 60% in other chips, such as sweet potato, beetroot and carrot crisps, by the end of intestinal digestion.^24^ Sansano *et al*.^25^ applied kinetic monitoring to assess the acrylamide elimination in potato chips and French fries during intestinal digestion. They showed that acrylamide elimination was rapid during the early phase then stabilized after 15 min of intestinal digestion.

**Table 2:** Changes in acrylamide content (μg kg^−1^) of potato chips and reformulated potato chips with cysteine and GSH during *in vitro* digestion

Sample	Initial	Gastric phase	Intestinal phase (bioaccessible)
Control	1749 ± 0.1^a^	1142 ± 134.9^b^	760 ± 177.3^c^
1% GSH	267 ± 2.4^a,^ *	227 ± 2.2^b^	264 ± 11.5^a^
0.5% Cysteine	227 ± 49.2^a,^ *	126 ± 5.7^b^	237 ± 10.8^a^

Results are expressed as mean ± SD. Different superscript letters indicate significant differences (*P* < 0.05) in the same row according to one‐way ANOVA Tukey test.

* Significant difference from the control according to Student's *t*‐test.

In contrast to elimination, the potential neo‐formation or release of acrylamide from the food matrix during digestion might be considered to evaluate dietary exposure. This concept was first reported by Hamzalıoğlu and Gökmen^4^ and was later independently verified by other researchers,^26^ who demonstrated that acrylamide levels may change under simulated digestion conditions due to matrix‐related effects or potential conversion of intermediates to acrylamide. This might be determined by analyzing acrylamide content in the insoluble part remaining from the bioaccessible fraction.^24^, ^26^

Hidalgo and others carried out studies investigating the Michael‐type reactions of both thiol and amino compounds with acrylamide.^27^, ^28^, ^29^ The decrease of acrylamide by interactions with nucleophiles under alkaline intestinal pH was also included in a review.^11^ To test this, the acrylamide adducts (Table 1) were monitored both in control, reformulated potato chips and their bioaccessible fractions after intestinal digestion. Elimination of the acrylamide in the bioaccessible fraction was found to be due to the interactions of amino compounds with acrylamide and released during digestion of the potato chips. HRMS analysis showed that arginine, aspartic acid, histidine, methionine, phenylalanine and proline adducts of acrylamide were detected in digested control samples, while they were not present prior to digestion. Li *et al*.^30^ synthesized and characterized a number of amino acid (glycine, lysine, tryptophan and GABA)–acrylamide adducts through Michael addition and reported their presence in baked foods and reducing effect on acrylamide cytotoxicity.

Incorporating thiol compounds (1% GSH or 0.5% cysteine) significantly lowered acrylamide content in potato chips (*P* < 0.05). The results pointed out that the addition of GSH and cysteine to the potato chip formulation at certain levels proved effective in reducing acrylamide levels in the reformulated potato chips to the levels below the benchmark levels set by the European Union during baking. In our previous study, we elucidated the inhibition mechanism as the competition of thiol compounds with asparagine for carbonyl compounds. This competition resulted in a reduced formation of the Schiff base intermediate of asparagine, ultimately leading to the decreased formation of acrylamide.^12^ However, the addition of thiol compounds into the chip formulation did not result in further elimination of acrylamide in the bioaccessible part (*P* > 0.05). This finding indicated that thiol compounds were effective at eliminating acrylamide during baking, but they had no further effect on its removal during digestion of reformulated chips. This might be due to the transformation of thiol compounds into their inactive oxidized forms during baking. GSH could transform into inactive forms during baking of reformulated chips. GSH is known to easily oxidize to GSSG.^31^ As monitored by HRMS, the oxidation of GSH to GSSG was noticeable in this study during both baking and digestion due to the thermal impact of baking and the subsequent pH changes (first acidic, then mildly alkaline throughout the gastric and intestinal phases) during digestion. Similarly, cysteine added to reformulated chips oxidized to cystine during baking. Another possible explanation for the absence of active cysteine or GSH in the potato chips, and consequently no further acrylamide reduction during digestion, is that both compounds might have competed with asparagine in the Maillard reaction during baking. As a result, no free cysteine or GSH might remain in the reformulated chips to react with acrylamide during digestion.

Previous studies reporting a decrease in acrylamide concentration during digestion only dealt with the food product itself, not reformulated with any thiol compounds or other compounds.^4^, ^26^ Our study examines real food matrices in which acrylamide is present and specific thiol compounds are deliberately incorporated in a controlled manner to evaluate their interaction under simulated digestion conditions. While the realistic meal‐based approach reported by González‐Mulero *et al*.^26^ demonstrated the contribution of endogenously occurring thiol groups from food components under simulated digestion conditions, the present experimental design is based on the controlled addition of selected thiol compounds to evaluate thiol–acrylamide and amino acid–acrylamide adduct formations. Rather than monitoring only changes in acrylamide concentration, we identify amino and thiol adducts formed during digestion (Table 1).

### Changes in acrylamide concentration during *in vitro* co‐digestion of potato chips with thiol compounds

The alterations in the reformulated chips during digestion indicated that no additional acrylamide removal took place during digestion, due to the transformations of precursors (cysteine and GSH) during baking. These findings suggest that further mitigation of acrylamide during digestion depends on the presence of active thiol compounds, which were no longer available in the baked matrix. A recent study demonstrated the effects that occur during digestion when acrylamide‐containing foods are consumed as part of a complete meal in combination with other foods.^26^ Those observations imply that co‐digestion with thiol compounds could be an effective method to mitigate the toxicity of acrylamide. For this reason, potato chips were digested together with free cysteine (0.5%) or GSH (1%) to stimulate potential acrylamide elimination during co‐digestion. The concentrations of thiols were selected based on their mitigation potential in acrylamide formation in reformulated chips and their sensorial properties.^12^ Using this specifically designed digestion model that simulates the concurrent dietary intake of acrylamide and thiols provides new insights into their interaction under realistic conditions. The free form of thiols was preferred for co‐digestion rather than a real food system containing thiol compounds, since our aim was to monitor the interactions of thiols with acrylamide present in the food system and avoid additional variables introduced by the matrix containing thiol compounds, which contains many other components that could hinder the specific effects under investigation. Since thiols are highly reactive in their free form, using them in this form allows for clear monitoring of their direct effects.

**Figure 2:**
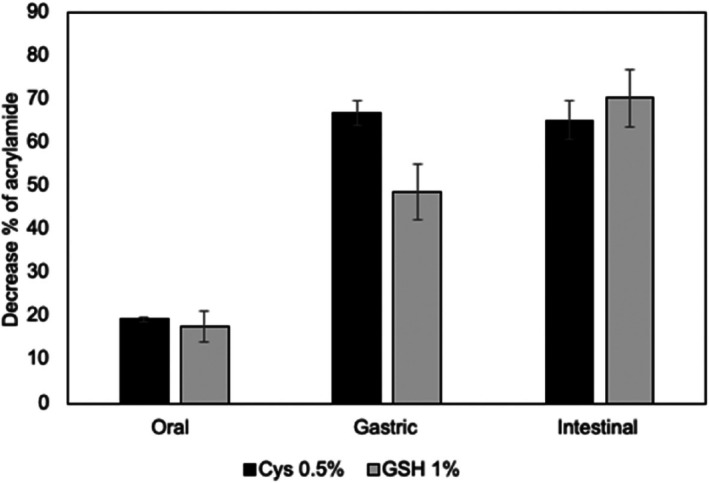
Decrease in acrylamide content in digesta from co‐digestion with 0.5% cysteine (Cys) and 1% glutathione (GSH) relative to control potato chips.

The concentration of acrylamide during co‐digestion of potato chips with thiols was evaluated relative to the initial acrylamide level in the chips, and the corresponding percentage reductions observed with 0.5% cysteine and 1% GSH are presented in Fig. 2. Thiol compounds, particularly cysteine, are highly reactive toward acrylamide. The acrylamide concentration exhibited a decreasing trend, compared to its initial concentration, even after the oral phase of co‐digestion. Acrylamide elimination was approximately 19% in the potato chip samples co‐digested with thiol compounds after the oral phase. In the co‐digestion experiments, acrylamide, a highly water‐soluble molecule, released rapidly from potato chips during the oral phase comes into immediate contact with free thiol compounds, potentially leading to its rapid elimination within 1–2 min.

After the gastric phase of co‐digestion with 1% GSH, 49% of acrylamide was eliminated, resulting in a final elimination rate of 70% at the end of the intestinal phase. As explained before, the possible reason might be the Michael addition of acrylamide to other amino acids. In the study of González‐Mulero *et al*., the trapping of acrylamide in potato‐based products was achieved through co‐consumption with protein‐rich foods.^26^ In that study, combined foods were prepared by including individual food items with protein‐rich foods in specified proportions in a meal, reflecting a typical meal composition. For example, French fries were combined with meat steak (beef) (3:2 French fries to meat steak) and Spanish food patatas‐pobre (potatoes cooked in oil at low temperature) was combined with scrambled eggs (1:1 patatas‐pobre to egg). Complete elimination of acrylamide was observed in the case of digestion of patatas‐pobre together with scrambled eggs. It is well known that egg is a good source of the cysteine‐rich protein ovalbumin.^32^ Such reduction was also observed with the consumption of meat together with French fries. It was suggested that the most probable reason is the Michael‐type addition reactions involved in trapping free acrylamide, which subsequently reduces its bioaccessibility. However, the study did not confirm the presence of adducts.

In our study, one way of eliminating acrylamide might result from the addition of acrylamide to GSH, by forming GSH‐Acr adduct with a molecular formula C_13_H_22_N_4_O_7_S. This adduct was confirmed in co‐digestion samples of reformulated potato chips with 1% GSH collected at the end, as analyzed by HRMS. The oxidized form of GSH, i.e. GSSG, was also found in all potato chip samples. GSH is naturally present in potatoes; however, even small amounts may be oxidized to GSSG during digestion, and its remaining concentration appears insufficient for adduct formation. Therefore, the addition of free form of GSH would increase the possibility of forming a GSH–acrylamide adduct and increased acrylamide elimination.

Co‐digestion of cysteine with acrylamide exhibited superior efficacy in eliminating acrylamide compared to co‐digestion of GSH (Fig. 2). An amount of 67% of acrylamide was eliminated after the gastric phase in co‐digestion of acrylamide with 0.5% cysteine. However, no significant difference (*P* > 0.05) was observed between the gastric and intestinal phases in potato chips co‐digested with 0.5% cysteine. A higher elimination ratio in acrylamide when it is co‐digested with cysteine could be attributed to the Michael‐type addition of cysteine to acrylamide, as free cysteine is available in the digestion medium. Acrylamide and cysteine may interact to form acrylamide–cysteine adducts (C_7_H_12_N_3_O_3_S). Additionally, the addition of two acrylamide molecules to cysteine (C_9_H_17_N_3_O_4_S) is also possible through both thiol and amino groups. These adducts were monitored by HRMS analysis in the co‐digestion samples including potato chips with 0.5% cysteine. The presence of acrylamide–cysteine adducts could not be confirmed in the co‐digestion samples collected at the end of the intestinal phase; nonetheless, the presence of the adducts of two acrylamide–cysteine was confirmed. Although cysteine was present as its oxidized form, i.e. cystine, rather than as free form, its reduction and formation of acrylamide adduct seem to be possible under simulated *in vitro* digestion conditions.

Formation of amino acid–acrylamide adducts might decrease the acrylamide toxicity. A recent study investigating the cell toxicity of acrylamide adducts with amino acids including cysteine reported that acrylamide adducts of cysteine were not toxic towards HCT‐19 and Caco‐2 cells. They also included that amino acid–acrylamide adduct formation was successful at mitigating the harmful effects of acrylamide, resulting in less reactive oxygen species generation in the intestinal lines, prevention of mitochondrial membrane depolarization and induction of autophagy.^33^

Overall, the findings suggested co‐digestion with thiol compounds, e.g. cysteine and GSH, might be a way to eliminate the toxicity of acrylamide during digestion. However, confirmation through *in vivo* studies is essential through monitoring acrylamide metabolites.

### Effects of reformulated potato chips on urinary AAMA and GAMA levels

While *in vitro* studies provide valuable insights under controlled conditions, they do not fully replicate the complexity of a living organism. To address this limitation and further evaluate the biological relevance of the findings of this study, *in vivo* experiments were performed. The acrylamide reduction effect of reformulated potato chips was investigated by analyzing acrylamide metabolites excreted in urine using a mouse model. A diet formulated with 15% control or thiol‐containing reformulated potato chips (1% GSH or 0.5% cysteine) was prepared and administered to mice for a one‐week period. Following that, urine samples were collected for subsequent analysis.

**Figure 3:**
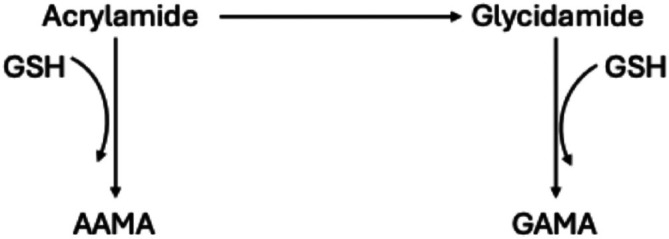
*In vivo* detoxification metabolism of acrylamide (adapted from Kocadağlı and Gökmen^35^). GSH, glutathione; AAMA, *N*‐acetyl‐*S*‐(2‐carbamoylethyl)‐l‐cysteine; GAMA, *N*‐(*R*,*S*)‐acetyl‐*S*‐(2‐carbamoyl‐2‐hydroxyethyl)‐l‐cysteine.

AAMA and GAMA are major indicative metabolites for acrylamide toxicity in urine (Fig. 3). Among early acrylamide metabolites, glycidamide serves as an indicator of metabolic toxification, whereas acrylamide–GSH may represent a promising biomarker of detoxification. Watzek *et al*.^34^ reported that the formation of GSH adducts from acrylamide occurs more rapidly than the formation of glycidamide in an *in vitro* study involving primary rat hepatocytes.

AAMA and GAMA levels obtained from urinary analysis are given in Table 3. The findings of this study demonstrated that the urinary levels of glycidamide in all urine samples of mice fed with control and reformulated potato chips were below the detection limit. This may be attributed to the conjugation of acrylamide with GSH, which plays a crucial role in limiting its metabolic conversion to glycidamide. The results are comparable to those of a previous study that investigated the toxicokinetic of acrylamide in six healthy young adults after consuming a 0.94 mg dietary acrylamide dose from potato chips. Urine samples were collected over a 72 h period, and the study reported that acrylamide detoxification via the formation of AAMA is more efficient than the formation of glycidamide.^36^

**Table 3:** Effects of reformulated potato chips on urinary AAMA and GAMA levels

Diet	Feed	Urine	
	Acrylamide (μg kg^−1^)*	AAMA (μg mL^−1^)	GAMA (μg mL^−1^)
Standard (*n* = 5)		0.0181 ± 0.002^c^	<LOD
Control chips (*n* = 7)	1155	0.0429 ± 0.006^b^	0.0451 ± 0.006^a^
1% GSH chips (*n* = 5)	315	0.0690 ± 0.016^a^	0.0208 ± 0.003^b^
0.5% Cysteine chips (*n* = 5)	200	0.0356 ± 0.002^b^	0.0154 ± 0.001^b^

* Acrylamide content of control chips: 7698 ± 384.9 μg kg^−1^; potato chips containing 1% GSH: 2101 ± 105.5 μg kg^−1^; potato chips containing 0.5% cysteine: 1331 ± 66.5 μg kg^−1^. The diets were incorporated with 15% chips. LOD for GAMA is 0.0016 μg mL^−1^. Different superscript letters indicate significant differences (*P* < 0.05) in the same column according to one‐way ANOVA Tukey test.

On the other hand, GAMA was below LOD in the urine of mice having a standard diet, whereas it was detected following the inclusion of potato chips in their diet. Similarly, AAMA was elevated in the control group fed with control potato chip diet in comparison to the standard group. In the 1% GSH group, AAMA levels increased by 60% while in contrast AAMA levels decreased by 17% in the 0.5% cysteine group. It is interesting to note that AAMA levels were increased in urine of mice fed GSH‐containing chips, despite the lower acrylamide exposure associated with those chips. The elevated AAMA levels may be associated with the metabolic pathway of acrylamide. During phase II biotransformation pathway, acrylamide conjugates with GSH, and the conjugate undergoes degradation and acetylation processes, resulting in the formation of AAMA.^37^ As 1% GSH was already present in reformulated potato chips, this metabolic process may have led to increased AAMA excretion. The decreased acrylamide content in 0.5% cysteine samples might be the reason behind the reduction in AAMA levels in the mice group fed with a 0.5% cysteine diet.

On the other hand, GAMA levels were less than half in urine samples of mice fed with thiol‐containing diet compared with mice fed with control diet. In the 1% GSH group, GAMA decreased by 54% whereas 66% of GAMA was eliminated in the 0.5% cysteine group (Table 3). GAMA is formed through the conjugation of glycidamide with GSH during phase II biotransformation pathway and is subsequently excreted in urine.^38^ The observed reduction in GAMA levels in urine might be due to the limited metabolic conversion of acrylamide to glycidamide, as previously discussed. In addition to this, the reductions could be attributed to the decreased acrylamide content in both GSH‐ and cysteine‐containing chips. These findings suggest that, in comparison to control potato chips, reformulated potato chips containing 1% GSH exhibit enhanced acrylamide detoxification effect.

## CONCLUSIONS

This study examined the impact of thiol compounds on the fate of acrylamide during *in vitro* digestion and its subsequent metabolism in C57BL/6N mice. An amount of 57% of acrylamide present in control chips was eliminated during digestion. This reduction was attributed to the interactions of amino compounds with acrylamide through Michael addition during digestion. The incorporation of GSH or cysteine resulted in a reduction in acrylamide formation during the baking process of reformulated potato chips; however, no further reduction was observed during digestion. The oxidation of GSH and cysteine during baking led to inactivation of their thiol group, resulting in no further interaction with acrylamide during digestion. Conversely, acrylamide elimination began at the oral phase when potato chips and free thiol source were consumed together. Co‐digestion further eliminated acrylamide during digestion, suggesting that co‐consumption could be a promising approach for eliminating acrylamide. The results from *in vivo* studies indicated that GSH‐containing chips enhanced acrylamide detoxification, as evidenced by increased urinary excretion of AAMA, while lowering GAMA levels, likely by limiting the conversion of acrylamide to glycidamide. Cysteine‐containing chips also lowered acrylamide exposure, as evidenced by decreased AAMA and GAMA levels. Collectively, these findings suggest that thiol compounds can effectively reduce dietary acrylamide exposure by both processing‐based reduction and, when present in free form during digestion, through elimination within the gastrointestinal tract. Reformulation with GSH or cysteine, or their co‐consumption with acrylamide‐rich foods, presents as a potential strategy to mitigate the potential toxicological effects of acrylamide.

## AUTHOR CONTRIBUTIONS

Burçe Ataç Mogol: Conceptualization, Methodology, Formal analysis, Investigation, Data curation, Visualization, Project Administration, Writing – original draft. Choong‐In Yun: Methodology, Formal analysis, Investigation, Data curation, Visualization, Writing – review and editing. Aytül Hamzalıoğlu: Conceptualization, Methodology, Formal analysis, Investigation, Data curation, Visualization, Writing – original draft. Selin Çakmak: Formal analysis, Investigation, Data curation. Yeşim Şensoy: Formal analysis, Investigation. Nazmiye Çitler: Formal analysis, Investigation. Eunjung Lee: Methodology, Formal analysis, Investigation. Shin Young Park: Methodology, Formal analysis, Investigation. Junwon Byun: Methodology, Formal analysis, Investigation. Young‐Jun Kim: Data curation, Writing – review and editing. Sanguine Byun: Conceptualization, Methodology, Resources, Supervision, Writing – review and editing. Vural Gökmen: Conceptualization, Methodology, Resources, Supervision, Writing – review and editing.

## CONFLICT OF INTEREST

The authors declare that they have no conflict of interest.

## Data Availability

Data will be made available on request to the author(s).
